# Corneal Endothelial Cell Density and Morphology in Healthy Egyptian Eyes

**DOI:** 10.1155/2019/6370241

**Published:** 2019-02-24

**Authors:** Marwa Mahmoud Abdellah, Hatem Gamal Ammar, Mohamed Anbar, Engy Mohammed Mostafa, Mahmoud Mohamed Farouk, Khulood Sayed, Alahmady Hammad Alsmman, Mohamed Gamal Elghobaier

**Affiliations:** ^1^Ophthalmology Department, Sohag University, Sohag, Egypt; ^2^Egyptian Police Hospital, Sohag University, Sohag, Egypt

## Abstract

**Purpose:**

To evaluate the corneal endothelial cell density and morphology in normal Egyptian eyes.

**Methods:**

In total, 568 healthy eyes of 568 Egyptian volunteers aged 20 to 85 years were examined using noncontact specular microscopy for the central corneal thickness (CCT), mean endothelial cell density (MCD), coefficient of variation (CV) in cell area, mean cell area (MCA), and hexagonal cell (Hex) percentage. Variables were compared between sexes and between different age groups.

**Results:**

The mean CCT, MCD, and MCA were 514.45 ± 43.04 *μ*m, 2647.50 ± 382.62 cells/mm^2^, and 390.59 ± 149.94 *μ*m^2^, respectively. MCD and MCA showed no significant differences between men and women (*P*=0.171 and 0.099, respectively), whereas CV (%) and Hex (%) showed significant differences (*P*=0.024 and 0.015, respectively). CCT (*P*=0.007, *r* = −0.113) and MCD (*P* < 0.001, *r* = −0.357) exhibited a significant negative correlation with age, whereas CV (%) (*P* < 0.001, *r* = 0.341) and MCA (*P*=0.008, *r* = 0.111) exhibited a significant positive correlation. The mean rate of endothelial cell loss from 20 to 85 years of age was 0.3% per year.

**Conclusions:**

Our results provide normative data for the corneal endothelium in healthy Egyptian eyes, thus increasing the knowledge base for corneal endothelial cell parameters in healthy Egyptian eyes. Furthermore, our findings can be used as baseline values for comparisons between Egyptian and other populations and for studies of the endothelial cell reserve and capacity for intraocular surgery and corneal transplantation.

## 1. Introduction

The corneal endothelium is formed by a single layer of cells on the posterior corneal surface and transports water from the stroma into the anterior chamber to preserve the corneal dehydration and its transparency [1]. In the past, assessment of the corneal endothelium was limited to biomicroscopic evaluations for guttae, folds, and keratic precipitates, and it was quite difficult to assess the endothelial cell characteristics and functional reserve [2, 3]. The introduction of advanced evaluation techniques, including specular microscopy, fluorophotometry, and pachymetry, has enabled evaluation of the corneal endothelial cell density and function.

In some species, the endothelium can regenerate after injury. On the contrary, humans exhibit a cell repair mechanism characterized by enlargement of the endothelial cells [1]. In the absence of the ability of endothelial cells to proliferate, the endothelial cover of the posterior corneal surface is maintained by a gradual increase in the size of the remaining cells, which result in increased cellular pleomorphism and a decrease in the percentage of hexagonal cells with age [4, 5].

Specular microscopy is used to establish and compare normative data for corneal endothelium parameters among different ethnic groups and between men and women [6–8]. In the present study, we aimed at evaluating the corneal endothelium cell density (CD) and morphology in healthy adult Egyptian eyes, determining changes in these variables of the endothelium with age, which is a major concern in intraocular surgeries, and comparing the findings with those for other ethnicities. To the best of our knowledge, this is the first study to report these data for the Egyptian population.

## 2. Materials and Methods

From 832 volunteers examined to be included by the study, 264 (31.73%) subjects were excluded; only 568 (68.27%) subjects adhered to our inclusion/exclusion criteria. The age of volunteers ranged from 20 to 85 years. Most of the subjects were healthy relatives of visiting patients or patients with transient ocular complaints, but no permanent eye pathologies. The exclusion criteria were as follows: a history of intraocular surgery, previous ocular trauma, increased intraocular pressure, uveitis, corneal opacity, evidence of endothelial dystrophy on slit lamp biomicroscopy, diabetes mellitus, ocular surface diseases, and contact lens wearers. The study is adherent to the guidelines of the Declaration of Helsinki (1964), it was approved by the Medical Ethics Committee of Sohag University, and a written informed consent was obtained from all volunteers after providing an explanation of the benefits of the study for research purposes.

First, routine ophthalmological examinations were performed, and if the participant was found to be eligible for the study, the corneal endothelium was examined using a noncontact specular microscope (Topcon SP-1P, Tokyo, Japan) with an autofocus device. Images of the endothelium were obtained on the incorporated screen provided with the device. The patients are asked to look to the fixation target; when the pupil is displayed, we taped the area around the pupil; the photographing head moves to display the pupil image and alignment dot on the center of the screen; alignment starts automatically, and photographing is performed; multiple images are taken by a central view; and once a clear image of a good quality of the central endothelium was captured, the centers of at least 100 contiguous endothelial cells were marked using software available in the system. A computer performed an automated analysis of the cell. Many images were taken but at least two images of a good quality of sufficient cell numbers and clear cell boundaries that appeared similar in their analyzed data were obtained to be sure of the accuracy. All examinations were done by the same researcher (Hatem Ammar) and by the same device. Parameters were displayed on the screen and obtained as a printout. The numbers of the cells should not be less than 100 cells in the center.

Parameters recorded from the system included the central corneal thickness (CCT), mean CD (MCD), mean cell area (MCA), coefficient of variation (CV) in cell area, and percentage of hexagonal cells (Hex (%)). CD was recorded as the number of cells per square millimeter. MCA and CV (standard deviation/MCA) were used as indices of variation in the cell area (polymegathism). Hex (%) in the analyzed area was used as an index of variation in the cell shape (polymorphism).

All variables were compared between men and women. Subjects were also divided by decade of age, starting from the third decade. Accordingly, six subgroups were formed (21–30 years, 31–40 years, 41–50 years, 51–60 years, 61–70 years, and >70 years).

All statistical analyses were performed using statistical package for social sciences (IBM-SPSS) software, version 24 (IBM; Chicago, USA). The results are expressed as means and standard deviations (SDs) for quantitative parameters and numbers and percentages for qualitative parameters. Student's *t*-test was used to compare mean values between the two groups, and one-way analysis of variance (ANOVA) (with least significant difference post hoc test) was used to compare mean values among more than two groups. The Mann–Whitney test was used instead of Student's *t*-test for nonparametric data. Pearson's chi square analysis was used to compare percentages. Pearson's correlation analysis was used to compare two quantitative variables. The value of *r* is explained as follows: *r* < 0.2, negligible correlation; 0.2–0.4, weak correlation; 0.4–0.7, moderate correlation; and 0.7–1, strong correlation. For all these tests, a *P* value of <0.05 was considered statistically significant, with *P* < 0.001 considered highly significant.

## 3. Results

The mean age of participants in this study was 49 ± 15.2 (range, 20–85) years. There was a slight female predominance: 267 men (47.0%) and 301 women (53.0%).

The number of right eyes was 294 (51.8%) and left eyes 274 (48.2%). The mean CCT was 514.45 ± 43.04 *μ*m (range, 445–662) (95% CI, 510.9–517.99). MCD was 2647.50 ± 382.62 cells/mm^2^ (range, 1093–3877) (95% CI, 2615.9–2679.03). Mean CV was 32.31% ± 5.08% (range, 16%–48%) (95% CI, 31.89%–32.73%). Mean Hex (%) was 53.79% ± 11.00% (range, 5%–81%) (95% CI, 52.89%–54.7%). MCA was 390.59 ± 149.94 *μ*m^2^ (95% CI, 378.233–402.95) (Table 1).

**Figure 1:**
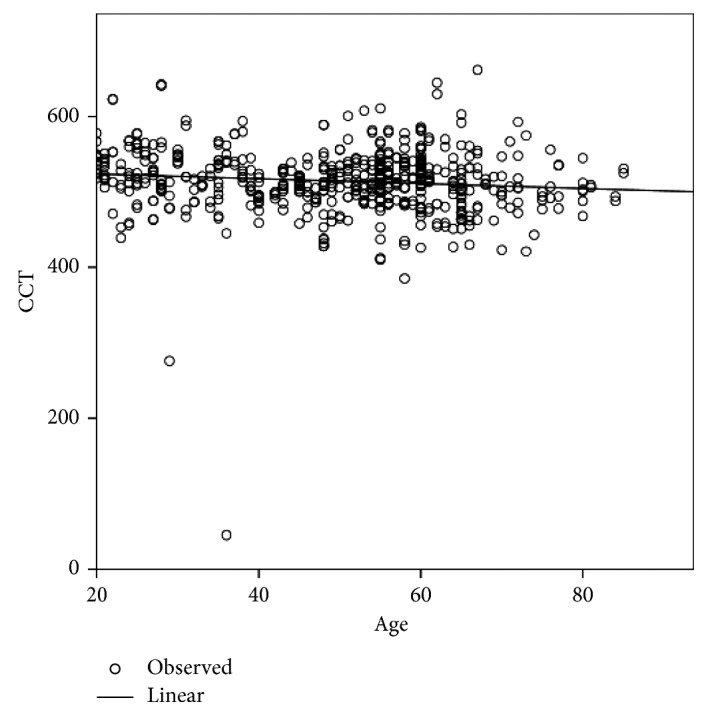
Relationship between the central corneal thickness (CCT) and age in Egyptian subjects with healthy eyes.

**Figure 2:**
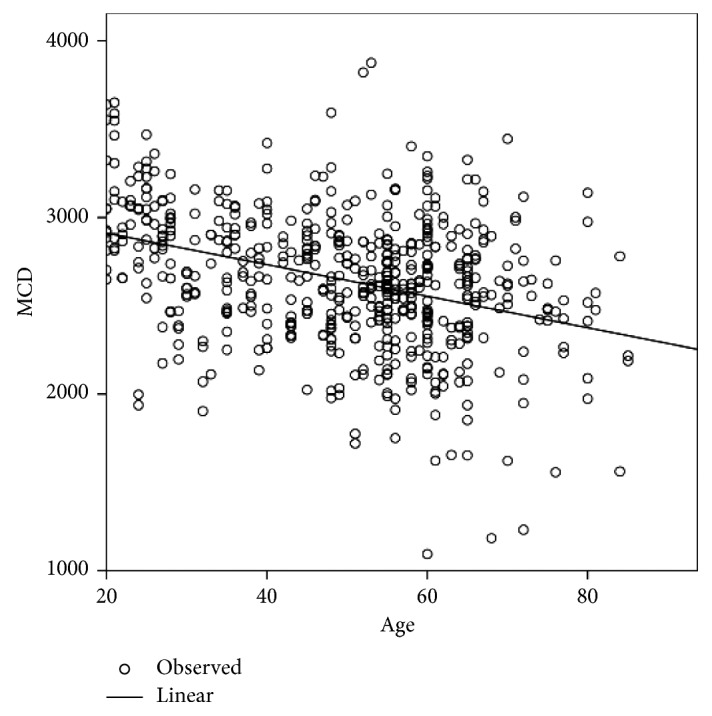
Relationship between the mean endothelial cell density (MCD) and age in Egyptian subjects with healthy eyes.

**Figure 3:**
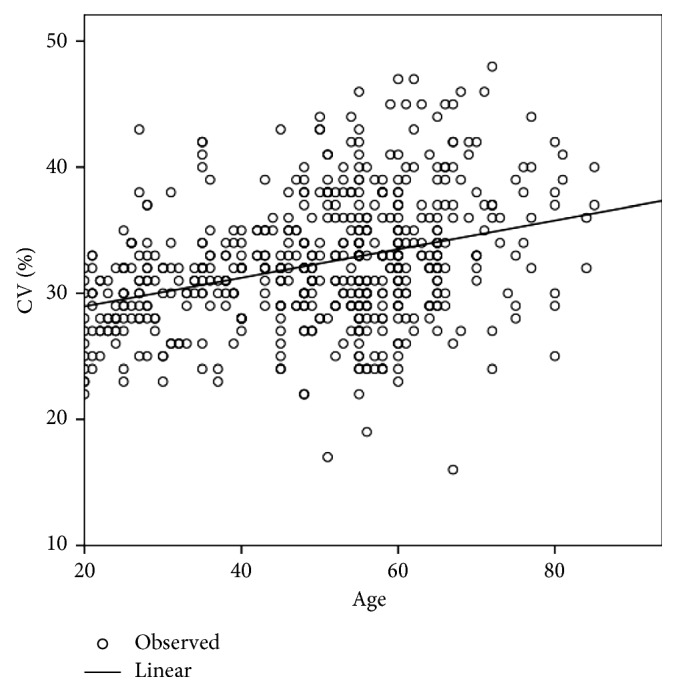
Relationship between the coefficient of variation (CV) in cell area and age in Egyptian subjects with healthy eyes.

**Figure 4:**
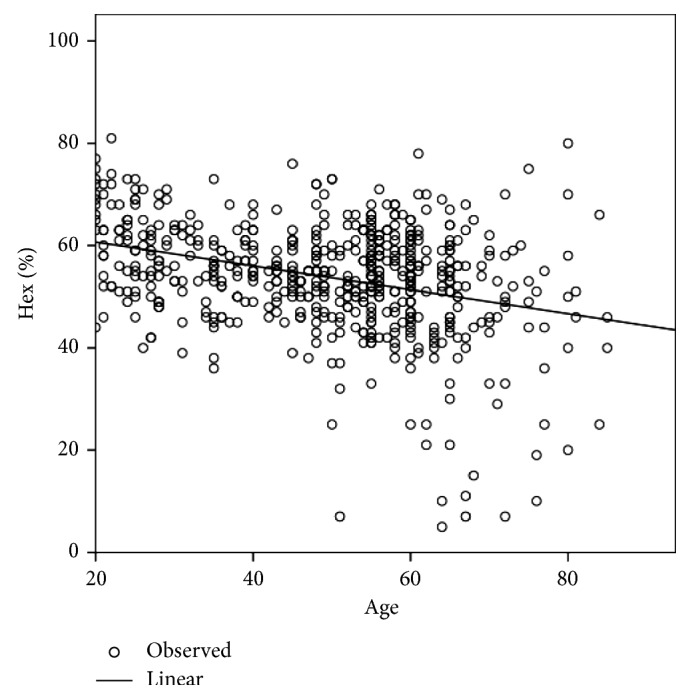
Relationship between the percentage of hexagonal cells (Hex (%)) and age in Egyptian subjects with healthy eyes.

**Figure 5:**
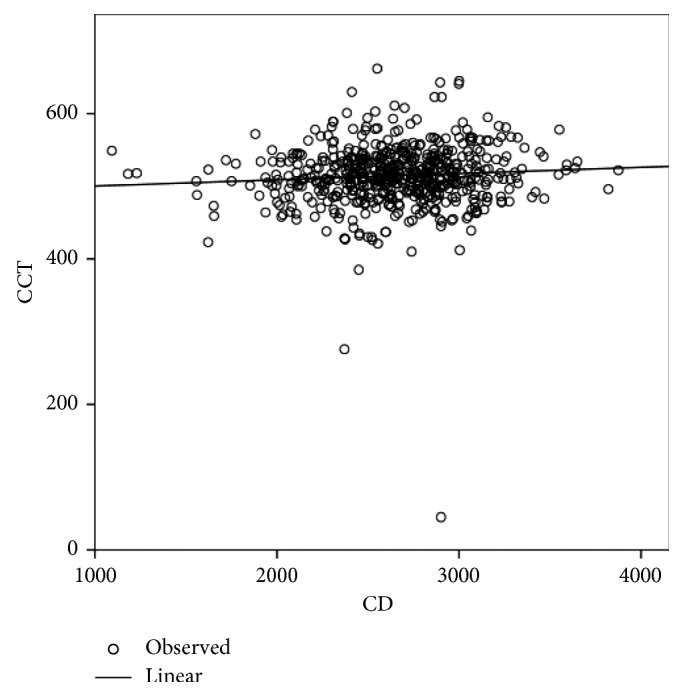
Relationship between the central corneal thickness (CCT) and the mean corneal endothelial cell density (MCD) in Egyptian subjects with healthy eyes.

Table 2 shows the differences in age and the corneal endothelial parameters between men and women. CV (%) and Hex (%) showed significant differences between men and women, whereas age, CCT, MCD, and MCA did not show any significant differences. The corneal endothelial cell density and morphology variables among different age groups of Egyptian subjects with healthy eyes are presented in Table 3. In the analysis of the different age groups, CCT, MCD, and Hex (%) were found to exhibit a significant negative correlation with age (Table 4; Figures 1–3), whereas CV and MCA were found to exhibit a significant positive correlation (Table 4; Figure 4). However, despite their significance, all correlations were found to be weak (*r* < 0.4). There was no significant correlation between CCT and MCD (*P*=0.069; Figure 5).

The mean corneal endothelial cell loss by decade in all subjects was 0.3%, and the higher endothelial cell loss rate 0.7% was in the age group 31–40 years (Table 5).

## 4. Discussion

In the present study, we evaluated the corneal endothelial cell density and morphology in normal Egyptian eyes using a Topcon noncontact specular microscope. The results revealed no significant differences in MCD between men and women. Furthermore, MCD significantly decreased with an increase in age, while MCA increased with age. A comparison with previous studies showed that MCD in our Egyptian population was lower than that in Japanese [7] and Chinese populations [9] and similar to that in American [7] and Nigerian populations [10]. It is worth to be mentioned that these studies also examined the endothelium in a wide age range and studied the difference in endothelium characteristics among different age groups as in this study.

The mean CCT was 517.06 ± 38.41 *μ*m. CCT decreased with an increase in age and was the highest in the 20–30-year age group (525.90 ± 47.65 *μ*m). This inverse correlation with age correlates with the study in Lithuanian population [11]. The CCT value obtained for Egyptians in the present study was lower than that obtained for Caucasian (550.4 *μ*m), Filipino (550.6 *μ*m), Hispanic (548.1 *μ*m), Japanese (531.7 *μ*m), and Latino (546.9 ± 33.5 *μ*m) populations and similar to that obtained for the African American population in previous studies [12, 13]. In a study of white European subjects [14], the mean CCT was 533 ± 0.033 *μ*m for adults and 527 ± 0.034 *μ*m for the elderly; both values were higher than the value for Egyptians obtained in the present study.

On comparison of the mean CCT for our subjects with that for subjects from southern Egypt [15], we found that the former was lower than the latter (530.06 ± 38.03 *μ*m). However, in the previous study, CCT was measured using ultrasonic pachymetry, not specular microscopy. This finding is in agreement with the findings in previous studies that confirmed lower CCT values obtained with specular microscopy than with ultrasonic pachymetry [16, 17].

Actually, CCT measurement differs among various devices of different principles of measurement. It was reported that the mean CCT acquired with noncontact specular microscopy in normal eyes ranged from 515 to 547 mm [18, 19], while the CCTs obtained by laser scanning confocal microscopy ranged from 520 to 563 mm [20]. Kiraly reported that CCT measurement by Cirrus HD-OCT is similar to IOL Master 700 and can be used interchangeably, and the both results are lower than Schiempflug imaging by Pentacam HR by about 10–12 *μ*m for the same patient [21].

While Faramarzi reported that the CCT measurements were the same by ultrasonic pachymetry and Orbsacn II [22], many papers agreed that specular microscopy had the poorest agreement with the other methods as the CCTs measured by specular microscopy were on average 20 to 30 mm thinner than those of the other methods [17, 23].

In most previous studies, CCT was found to decrease with an increase in age [12, 13]. A significant negative association between CCT and age was also found in the present study (*r* = −0.113, *P*=0.007). The mean CCT values for men and women were 517.06 ± 38.41 and 512.13 ± 46.71 *μ*m in our study, with no significant difference between sexes. This finding was also in concordance with the findings in other studies [24–26].

In the present study, the relationship between CCT and ECD was not significant (*P* < 0.069), as opposed to the finding of Nieider et al., who found a negative correlation between CCT and the endothelial cell count [27], while Galgauskas reported that ECD and CCT correlated directly, linking high ECD with thick corneas [11].

We also assessed the corneal endothelium for different age groups in our study of healthy Egyptian eyes. Several studies have reported the relationship of the corneal endothelial cell density and morphology with age, sex, and ethnicity. The relationships of age and sex with the characteristics of the corneal endothelium were found to differ among nations, which indicate that the corneal endothelial characteristics differ among races and ethnicities [6–9,28].

MCD in men and women in our study was 2670.9 ± 343.1 cells/mm^2^ and 2626.8 ± 414.0 cells/mm^2^, respectively, with no significant difference between sexes. With the exception of a study including healthy Chinese eyes, where MCD was significantly higher in men than in women [9], and a study including Filipino eyes, where MCD was significantly higher in women than in men [6], all previous studies have reported an insignificant difference in MCD between men and women [29–32]. In the study of Filipino eyes, it was reported that women are less susceptible to aphakic and pseudophakic corneal edema because of the higher MCD [6]. In the present study, MCD was 2933.75 ± 345.920 cells/mm^2^ for the 20–30-year age group and 2456.81 ± 443.648 cells/mm^2^ for the >70-year age group. The value for the older age group indicates a good endothelial reserve for intraocular surgeries, mainly for senile cataract. The mean annual rate endothelial cell loss in the present study was 0.3%, which is similar to that described in other cross-sectional studies (0.3%–0.5% per year) using a similar method of linear regression analysis [28, 31, 32]. However, the annual rate of cell loss differed among the age groups, with the highest rate of 0.7% observed for the 21–30-year group. Although the exact reason is not known, possible reasons include higher levels of physical activity and indirect eye trauma in this age group. This could also be a physiological phenomenon observed at this age, considering it has been noted in previous studies of different ethnicities [7, 28].

On comparison of MCD values for different age groups among American [7], Japanese [7], Indian [8], Chinese [9], Nigerian [10], Lithuanian [11], Spanish [33], and Egyptian populations (Table 6), we found that the value for Egyptians was significantly higher than that for Indians, significantly lower than those for the Japanese and Chinese, and similar to that for Americans. With comparison to European nations, Egyptian are quite similar to Lithuanian population but lower than Spanish population in young age group (20–40 years) and approach Spanish population values above fifty years. The value for individuals aged 41–50 years was higher in our Egyptian population than in the Nigerian population, whereas that for individuals aged >70 years was much lower for our Egyptian population than for the Nigerian population [7–11,33].

Mastuda et al. speculated that the corneal diameter is inversely proportional to the endothelial cell count, and this is the main reason for the significantly lesser CD in Americans than in the Japanese [7]. Furthermore, Rao et al. found that the corneal diameter in Indians was larger than that in Americans and the Japanese, which explains the lower MCD in Indians than in the other two populations [8]. In the present study, however, we did not measure the corneal diameter to investigate this theory.

The human corneal endothelium comprises cells with varying surface areas. The polymegathism value is a coefficient describing the variation in cell area [34]. In the present study, CV showed a significant positive correlation with age. In a Filipino study [6], it was found that polymegathism was greater in men (CV, 32.82% ± 5.00%) than in women (31.85% ± 5.12%; *P*=0.024). On the other hand, studies including Iranian and Turkish populations found no significant differences in CV between sexes [26, 35].

Finally, Hex (%) showed a significant negative correlation with age; a finding similar to those in previous studies [6, 9]. However, there was a significant difference in Hex (%) between our Egyptian men and women, whereas no significant difference between sexes was found in other studies [19, 27]. The significant difference in CV and Hex (%) between men and women in Egypt could be attributed to the effect of smoking on corneal endothelium. Smoking is prevalent among Egyptian men much more than Egyptian women [36]. This assumption was tested by Sayin et al. who revealed a lower percentage of endothelial hexagonal cells observed in smokers than nonsmokers (*P* < 0.001) [37] and Ilhan et al. who found decreased ED and Hex, with increased CV in smokers versus nonsmokers [38].

## 5. Conclusion

To the best of our knowledge, this is the first report of endothelial cell characteristics in healthy Egyptian eyes. Our results provide normative data for the corneal endothelium in healthy Egyptian eyes, thus increasing the knowledge base for corneal endothelial cell parameters in healthy Egyptian eyes. Furthermore, our findings can be used as baseline values for comparisons between Egyptian and other populations and for studies of the endothelial cell reserve and capacity for intraocular surgery and corneal transplantation.

## Figures and Tables

**Table 1:** Demographic characteristics and variables pertaining to the corneal endothelial cell density and morphology for Egyptian subjects with healthy eyes.

Value (%)		Variable
Sex	Male	267 (47.0%)
	Female	301 (53.0%)

Age	20–30 years	89 (15.7%)
	31–40 years	65 (11.4%)
	41–50 years	96 (16.9%)
	51–60 years	153 (26.9%)
	61–70 years	122 (21.5%)
	>70 years	43 (7.6%)
	Mean ± SD	49.48 ± 15.27
	Median (range)	53 (18–85)

Eye	Right eye	294 (51.8%)
	Left eye	274 (48.2%)

CCT (*μ*m)	Mean ± SD	514.45 ± 43.04
	Median (range)	513 (45–662)
	95% CI	510.9–517.99

MCD (cells/mm^2^)	Mean ± SD	2647.50 (382.62)
	Median (range)	2652 (1093–3877)
	95% CI	2615.9–2679.03

CV in cell area (%)	Mean ± SD	32.31 ± 5.08
	Median (range)	32 (16–48)
	95% CI	31.89–32.73

Hex (%)	Mean ± SD	53.79 ± 11.00
	Median (range)	55 (5–81)
	95% CI	52.89–54.7

MCA (*μ*m^2^)	Mean ± SD	390.59 ± 149.94
	Median (range)	376 (50.3–3489)
	95% CI	378.233–402.95

SD: standard deviation; CCT: central corneal thickness; MCD: mean endothelial cell density; CV: coefficient of variation; Hex: hexagonal cells; MCA: mean cell area; CI: confidence interval.

**Table 2:** Comparison of age and variables pertaining to the corneal endothelial cell density and morphology between men and women in our cohort of Egyptian subjects with healthy eyes.

Item	Men (mean ± SD)	Women (mean ± SD)	*t*-Test	*P* value
Age	49.60 ± 15.39	49.37 ± 15.18	0.177	0.860
CCT (*μ*m)	517.06 ± 38.41	512.13 ± 46.71	1.364	0.173
MCD (cells/mm^2^)	2670.9 ± 343.1	2626.8 ± 414.0	1.372	0.171
CV in cell area (%)	32.82 ± 5.00	31.85 ± 5.12	2.270	0.024
Hex (%)	52.60 ± 11.43	54.85 ± 10.51	2.473	0.015
MCA (*μ*m^2^)	392.23 ± 199.91	389.13 ± 83.95	36961.5	0.099

SD: standard deviation; CCT: central corneal thickness; MCD: mean endothelial cell density; CV: coefficient of variation in cell area; Hex: hexagonal cells; MCA: mean cell area.

**Table 3:** Correlation between age and variables pertaining to the corneal endothelial cell density and morphology in Egyptian subjects with healthy eyes.

	Pearson's correlation coefficient (*r*)	*P* value
CCT (*μ*m)	−0.113	0.007
MCD (cells/mm^2^)	−0.357	<0.001
CV in cell area (%)	0.341	<0.001
Hex (%)	−0.323	<0.001
MCA (*μ*m^2^)	0.111	0.008

CCT: central corneal thickness; MCD: mean endothelial cell density; CV: coefficient of variation in cell area; Hex: hexagonal cells; MCA: mean cell area.

**Table 4:** Comparison of variables pertaining to the corneal endothelial cell density and morphology among different age groups of Egyptian subjects with healthy eyes.

Age group (years)		Age	CCT (*μ*m)	MCD (cells/mm^2^)	CV in cell area (%)	Hex (%)	MCA (*μ*m^2^)
20–30	Mean	24.44	525.92	2933.75	29.44	60.21	345.303
	SD	2.884	46.83	345.92	3.56	9.02	55.96
	95% CI		517.2–536.8	2843.5–2983.7	28.6–30.1	58.3–61.9	336.5–358.8

31–40	Mean	34.83	518.65	2693.57	31.05	54.85	416.77
	SD	2.81	67.99	287.34	4.21	7.490	391.56
	95% CI		496.7–527.2	2638.9–2788.7	30.3–32.1	53.3–56.9	323.3–498.5

41–50	Mean	45.36	503.62	2663.65	31.74	54.92	379.896
	SD	2.96	28.57	332.18	3.83	7.12	48.27
	95% CI		500.1–512.7	2591.3–2720.4	31.4–33.2	52.5–55.9	370.8–389.9

51–60	Mean	54.80	516.92	2582.88	32.65	53.75	391.616
	SD	2.39	34.56	333.09	5.49	9.42	56.46
	95% CI		512.5–522.6	2532.9–2636.2	31.7–33.3	52.3–54.8	383.4–400.9

61–70	Mean	63.02	512.59	2549.68	33.98	50.17	404.615
	SD	2.74	42.71	414.12	5.45	13.17	82.33
	95% CI		498.4–517.3	2430.2–2606.5	33.4–35.8	46.1–52.2	393.7–439.1

>70	Mean	75.42	504.98	2456.81	35.47	46.81	425.186
	SD	4.61	35.33	443.65	5.29	16.17	139.68
	95% CI		495.1–518.7	2287.2–2577.3	33.8–37.5	40.5–52.1	380.6–454.8

SD: standard deviation; CCT: central corneal thickness; MCD: mean endothelial cell density; CV: coefficient of variation; Hex: hexagonal cells; MCA: mean cell area; CI: confidence interval.

**Table 5:** Corneal endothelial cell loss in different age groups of Egyptian subjects with healthy eyes.

Corneal endothelial cell loss by decade		
Age group (years)	Mean endothelial cell density (cells/mm^2^) (mean ± SD)	Rate of cell loss (%)
20–30	2933.75 ± 343.92	
31–40	2693.57 ± 287.35	0.7
41–50	2663.65 ± 332.18	0.1
51–60	2582.88 ± 333.09	0.3
61–70	2549.680 ± 414.15	0.1
71–80	2456.81 ± 44.3.65	0.3

**Table 6:** Comparison of the mean corneal endothelial cell density according to age among healthy Egyptian, Indian, American, Japanese, Nigerian, Chinese, Lithuanian, and Spanish eyes.

Age group (years)	Cell density (cells/mm^2^) (mean ± SD)							
	Egyptian population	Indian population	American population	Japanese population	Nigerian population	Chinese population	Lithuanian population	Spanish population
20–30	2933.19 ± 343	2782 ± 250	2977 ± 324	3893 ± 259	2861 ± 227	2988 ± 243	2931 ± 371	3233 ± 113
31–40	2693.57 ± 287	2634 ± 288	2739 ± 208	3688 ± 245	2631 ± 394	2920 ± 325	2820 ± 203	3033 ± 64
41–50	2663.65 ± 332	2408 ± 274	2619 ± 321	3749 ± 407	2434 ± 442	2935 ± 285	2660 ± 301	2854 ± 51
51–60	2582.88 ± 333	2438 ± 309	2625 ± 172	3386 ± 455	2545 ± 320	2810 ± 321	2630 ± 306	2577 ± 134
61–70	2549.68 ± 414	2431 ± 357	2684 ± 384	3307 ± 330	2538 ± 362	2739 ± 316	2518 ± 281
>70	2456.81 ± 444	2360 ± 357	2431 ± 339	3298 ± 313	2610 ± 371	2778 ± 365	2341 ± 167

## Data Availability

The data used to support the findings of this study are available from the corresponding author upon request.
